# Assessment of Pollution and Health Risks of Heavy Metals in Particulate Matter and Road Dust Along the Road Network of Dhanbad, India

**DOI:** 10.5696/2156-9614-11.29.210305

**Published:** 2021-03-02

**Authors:** Shweta Kumari, Manish Kumar Jain, Suresh Pandian Elumalai

**Affiliations:** Department of Environmental Science and Engineering, Indian Institute of Technology (Indian School of Mines), Dhanbad, Jharkhand -826004 (India)

**Keywords:** particulate matter, road dust, principal component analysis, health risk assessment

## Abstract

**Background.:**

The rise in particulate matter (PM) concentrations is a serious problem for the environment. Heavy metals associated with PM_10_, PM_2.5_, and road dust adversely affect human health. Different methods have been used to assess heavy metal contamination in PM_10_, PM_2.5_, and road dust and source apportionment of these heavy metals. These assessment tools utilize pollution indices and health risk assessment models.

**Objectives.:**

The present study evaluates the total mass and average concentrations of heavy metals in PM_10_, PM_2.5_, and road dust along selected road networks in Dhanbad, India, analyzes the source apportionment of heavy metals, and assesses associated human health risks.

**Methods.:**

A total of 112 PM samples and 21 road dust samples were collected from six stations and one background site in Dhanbad, India from December 2015 to February 2016, and were analyzed for heavy metals (iron (Fe), lead (Pb), cadmium (Cd), nickel (Ni), copper (Cu), chromium (Cr), and zinc (Zn)) using atomic absorption spectrophotometry. Source apportionment was determined using principal component analysis. A health risk assessment of heavy metal concentrations in PM_10_, PM_2.5_, and road dust was also performed.

**Results.:**

The average mass concentration was found to be 229.54±118.40 μg m^−3^ for PM_10_ and 129.73 ±61.74 μg m^−3^ for PM_2.5_. The average concentration of heavy metals was found to be higher in PM_2.5_ than PM_10_. The pollution load index value of PM_10_ and PM_2.5_ road dust was found to be in the deteriorating category. Vehicles were the major source of pollution. The non-carcinogenic effects on children and adults were found to be within acceptable limits. The heavy metals present in PM and road dust posed a health risk in the order of road dust> PM_10_> and PM_2.5_. Particulate matter posed higher health risks than road dust due to particle size.

**Conclusions.:**

The mass concentration analysis indicates serious PM_10_ and PM_2.5_ contamination in the study area. Vehicle traffic was the major source of heavy metals in PM_10_, PM_2.5_, and road dust. In terms of non-carcinogenic risks posed by heavy metals in the present study, children were more affected than adults. The carcinogenic risk posed by the heavy metals was negligible.

**Competing Interests.:**

The authors declare no competing financial interests

## Introduction

Particulate matter (PM) contamination is an important issue for the environment. Particulate matter is made up of solid and liquid components which can absorb compounds such as polycyclic aromatic hydrocarbons (PAH), heavy metals, elemental carbon, soot, acid salts, sulfate (SO^2^_4_) and nitrate (NO_3_) and allergens which encourage gene mutation and finally lead to cancer.^1,2^ Generally, PM can be categorized into two classes based on particle size; PM_10_ (particles with an aerodynamic diameter of ≤10 μm) and PM_2.5_ (particles with an aerodynamic diameter of ≤2.5 μm). Road dust acts as a temporary sink of metals coming from a variety of sources and as a source of pollutants contributing to atmospheric pollution through resuspension.^3,4^ In urban areas, road dust acts as a sink for pollutants, and PM gets deposited to the surface of the road in the form of road dust mainly due to forces which include gravity, particle drag, Brownian diffusion, electrical charge effects and particle inertia.^5,6^ Deposition and suspension are the collective effects of all of these forces that yield sufficient particle velocities in the downward and upward direction in the atmosphere. 7 Sources of PM include the incomplete combustion of fuel, coal mining and mining-related processes, trash burning, construction, transportation, and burning of agricultural produce. Transportation and vehicle traffic emitting PM are considered to be the primary sources of these pollutants in urban areas.^8–11^ Industrialization and urbanization are the major reasons for the rapid increase in the number of vehicles, which causes a higher PM load in the urban atmosphere.^12^

Particulate matter causes poor air quality, loss of visibility, climate change, and also influences radiative forces.^13,14^ Heavy metals attached to PM in ambient air and road dust have become a significant concern and pose a higher risk to human health.^15–17^ Particulate matter size plays an important role in heavy metal accumulation and its effects. Finer particles have a greater surface area, and therefore they accumulate heavy metals more effectively than coarser PM.^18^ Although a minute fraction of heavy metals contribute to PM, even this minute fraction can lead to significant and severe human health effects by inhalation, ingestion, and dermal absorption.^19,20^ Heavy metals have a tendency to become bio-accumulated through the food chain.^21^ This bioaccumulation causes cardiotoxicity, neurotoxicity, immune toxicity, and cancer, which results in an increased mortality rate.^17^ Because of the harmful and toxic effects caused by heavy metals in urban areas, the determination of heavy metal concentrations in atmospheric PM and road dust and analysis of its associated health risks is essential. Similar studies have been done in India,^22–28^ China,^29–33^ and other parts of the world.^6,34–40^

The main objectives of the present study were to assess heavy metal pollution present in ambient PM and road dust, examine possible sources, and determine its potential environmental impacts and health risks to those residing along the traffic roadways in Dhanbad, India in the winter season. The results can form the basis of policies to reduce urban pollution and improve public health.

**Abbreviations**
*CF*: Contamination factor
*EF*: Enrichment factor
*I_geo_*: Geo-accumulation index
*mCd*: Modified degree of contamination
*PI*: Pollution load index
*RI*: Risk index

## Methods

**Figure 1:**
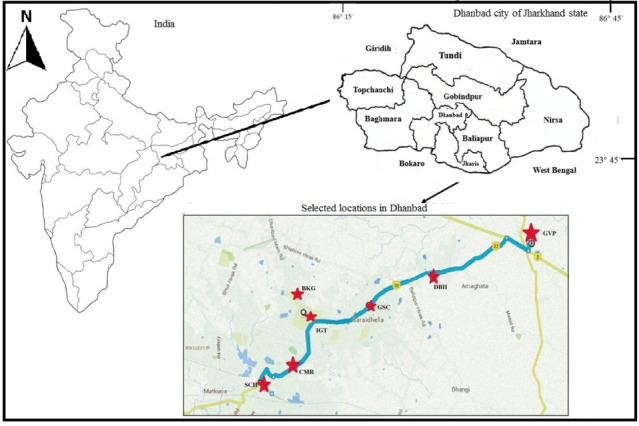
Map of the Dhanbad area and sampling sites

The study area, Dhanbad, India, is located in the state of Jharkhand and is one of the most critically polluted areas in India according to the Central Pollution Control Board (CPCB).^41^ It is located between 23°46′03″ N and 86°17′30″E. The major activities in Dhanbad are coal mining and associated activities such as commercial activities along the roadway and road transportation including private vehicles.^42^ The high flow of traffic is also a significant contributor to PM pollution.^23,43^ In 2019, there were a reported 408 000 registered vehicles in the city.^44^ The present study aimed to analyze PM along National Highway 18 (NH-18), which is the most essential road network for the city of Dhanbad. A 12-km-long segment of road was selected where no industrial and mining activities occur. Six sampling sites from Shramik Chowk to Govindpur *(Figure 1)* and one background site were selected according to the guidelines on site selection standards provided by IS: 5182 Part XIV (BIS, 2000).^45^ The details of the studies locations are presented in *Table 1*.

**Table 1:** Location Details of the Selected Sampling Sites

Location	Location Code	Geographical Location
Shramik Chowk	SCH	23°47′39″N 86°25′31″E
Court more	CMR	23°47′54″N 86°26′14″E
Indian Institute of Technology (Indian School of Mines) main gate	IGT	23°48′33″N 86°26′32″E
Gurukulam school	GSC	23°48′59″N 86°27′54″E
Dainik Bhaskar	DBH	23°49′31″N 86°29′10″E
Govindpur	GVP	23°50′08″N 86°30′53″E
Indian Institute of Technology (Indian School of Mines) Department of Environmental Science and Engineering	BKG	23°48′45″N 86°26′24″E

### Sample collection

Samples of PM_10_, PM_2.5_, and road dust were collected from six selected sites along with one background site in the winter season from December 2015 to February 2016.

Ambient air samples were collected for 24 hours twice a week (from December 2015 to February 2016). For collection of PM, the instruments were kept at a breathing level of 6 feet above the ground as per the standard monitoring guideline. A total of 112 PM (PM_10_ and PM_2.5_) samples were collected from the selected locations (total 56 PM_10_ samples and 56 PM_2.5_ samples, 8 samples from each location). The PM_10_ samples were collected on Whatman grade paper (EPM 2000 8″ × 10″) using a respirable dust sampler (Envirotech APM 460 NL) at a flow rate of 1.1 to 1.26 m^3^ min^−1^. The PM_2.5_ samples were collected using a fine particulate sampler (Envirotech APM 550 MFC) with a flow rate of 16.7 liters per minute on polytetrafluoroethylene (PTFE) filter papers of 47 mm diameter. The instruments were calibrated before sampling for proper measurement of PM_10_ and PM_2.5_. The initial and final weights of filter papers were taken using a microbalance (manufactured by AND HR-200) and the differences were recorded to calculate PM_10_ and PM_2.5_ concentrations. Filters were selected after carrying out pin hole inspection to avoid sampling error. These selected filter papers were conditioned in desiccators for 24-hr before and after sample collection. In addition, field blank filters and lab blanks were carried to and from the field to minimize the gravimetric error due to filter handling. After sampling, the samples were kept in polyethylene bags and stored at 4°C in a refrigerator. The mass concentration of PM was calculated by Equation 1.


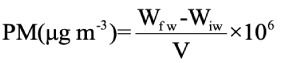


Where W_fw_ =final weight of filter paper (g), W_iw_ = initial weight of filter paper (g) V = total air volume (m^3^) 10^6^ = conversion of g to μg.

The road dust samples from the road surface of the selected sites and the background site were collected in the winter season by carefully sweeping them with a dust-free plastic brush in a dustpan. An area of 1 m × 1 m was swept to collect a sufficient amount of sample. A total of 21 samples (six sites × three samples per sites) were collected and kept in a clean polyethylene zip-lock bag and transported to the laboratory. All the collected samples were dried in an oven at a temperature of 110°C for 48 hours. These oven-dried samples were sieved using size 200 mesh sieves and kept in zip-lock bags.

### Sample preparation for heavy metal determination in PM_10_, PM_2.5_, and road dust

The heavy metals present in the PM and road dust samples were determined by atomic adsorption spectroscopy (AAS). For heavy metal determination, the samples were acid digested, the details of which are described in Supplemental Material Text S1 and Text S2, respectively.

### Statistical analysis

Principal component analysis was used to establish the probable sources and explain the relationship between heavy metals in PM_10_, PM_2.5_ and road dust in the study area.^46,47^ Principal component analysis with varimax rotation and Kaiser normalization was performed using the Statistical Package for the Social Sciences (SPSS) software (version 21.0). In the present study, principal components having the eigenvalues greater than 1 were extracted^48^ using the heavy metal concentration of all 112 samples (56 samples from each type; PM_10_ and PM_2.5_).

### Contamination assessment

The EF (enrichment factor), geo-accumulation index (Igeo), contamination factor (CF), pollution load index (PLI), modified degree of contamination (mCd), and risk index (RI) were used to evaluate the contamination level of PM_10_, PM_2.5_ and road dust samples. The details of these contamination assessment tools are given in Supplemental Material Text S3.

### Human health risk assessment

The extent and risks of exposure of heavy metals present in PM_10_, PM _2.5_, and road dust to human health were calculated using the United States Environmental Protection Agency (USEPA) human health evaluation method (USEPA, 2001).^49^ Exposure may occur via any of three paths: ingestion and deposition of PM and road dust, inhalation of atmospheric PM and road dust, and dermal absorption through the skin. The details of the human health risk assessment methods are given in Supplemental Material Text S4.

## Results

**Figure 2:**
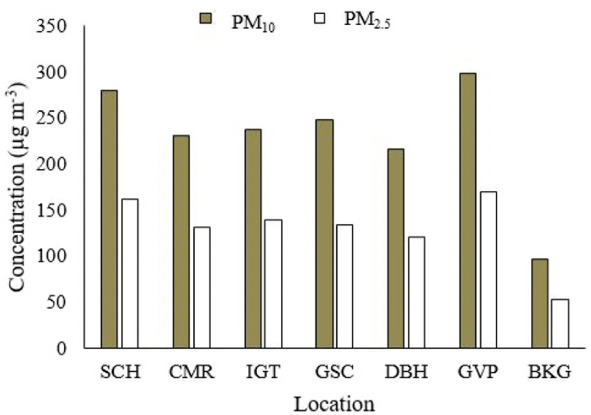
Average PM concentration at selected locations

The average mass concentration of PM_10_ and PM_2.5_ is shown in *Figure 2*. The average concentration of PM was highest at GVP (PM_10_ 298.31 μg m^−3^ and PM_2.5_ 169.68 μg m^−3^) and lowest at DBH (PM_10_ 215.69 μg m^−3^ and PM_2.5_ 120.51 μg m^−3^).

### Heavy metal analysis of PM_10_, PM_2.5_ and road dust

**Figure 3:**
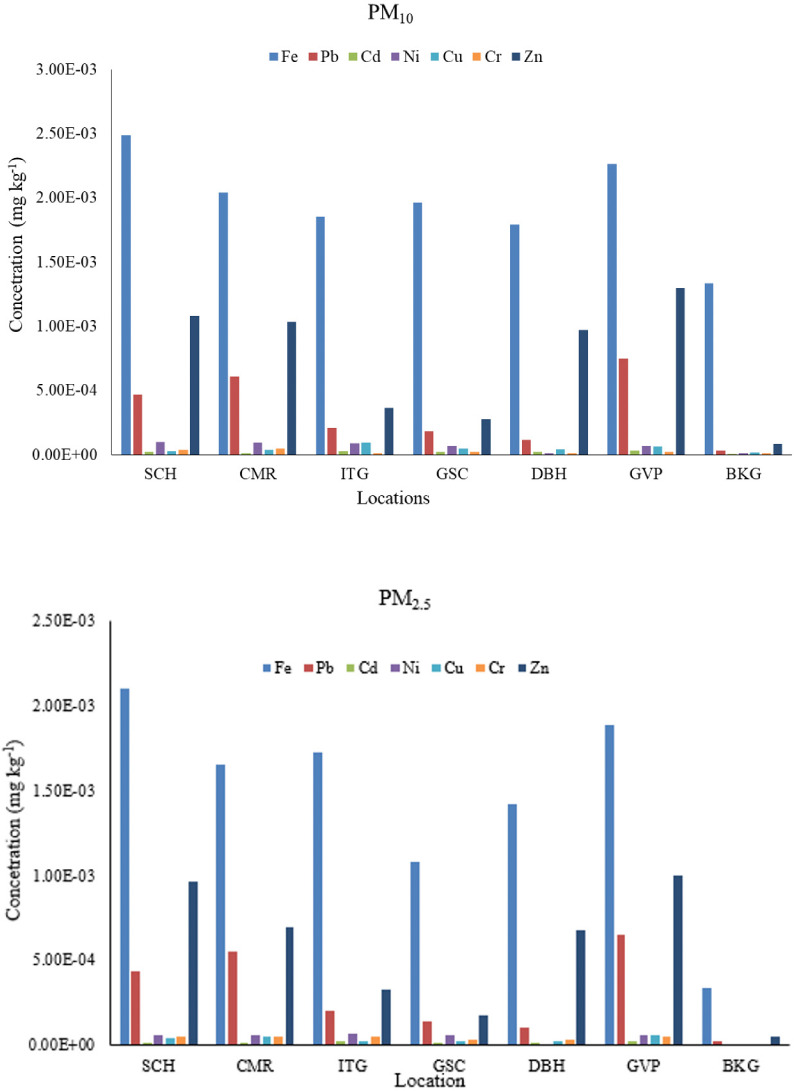
Mean heavy metal concentrations in PM_10_ and PM_2.5_ across study sites

**Figure 4:**
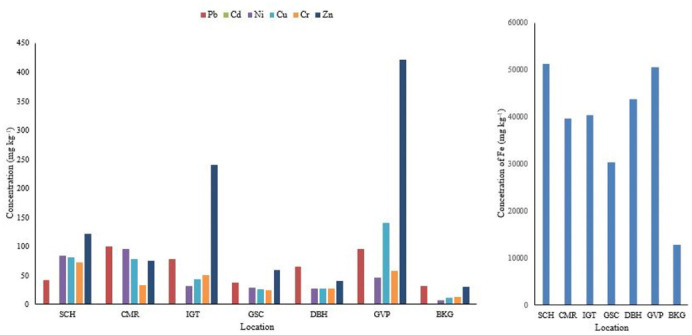
Mean heavy metal concentrations in road dust across study sites

Analysis of heavy metals is important to evaluate the health risk, contamination level, and emission sources linked to traffic, industrial, and residential activities. The present study found variation in the concentration of selected heavy metals (iron (Fe), lead (Pb), cadmium (Cd), nickel (Ni), copper (Cu), chromium (Cr), and zinc (Zn)) present in PM_10_, PM_2.5_, and in road dust samples across the study locations. The average values of the heavy metals in the samples of PM_10_ and PM_2.5_ across selected areas are given in *Figure 3*. The mean concentrations of heavy metals in road dust across locations are shown in *Figure 4*.

### Principal component analysis

Principal component analysis was performed to determine the possible contribution of sources to the heavy metals present in PM_10_, and PM_2.5_, and road dust. The area near the roadway was a busy location with heavy traffic. Additionally, different activity sites such as automobile repair shops, petrol pump, railway station, hospitals, hotels, restaurants, photocopy shops, tea shops, welding shops, paper printing press, and coal burning were dominant.

### Source identification of heavy metals in PM_10_, PM_2.5_, and road dust

The correlation coefficient of heavy metals in PM_2.5_ showed the positive correlation between Fe-Pb (0.668), Fe-Cr (0.805), and Fe-Zn (0.736), Pb-Cu (0.943), Pb-Cr (0.715), and Pb-Zn (0.651). A positive correlation was also observed between Ni-Cr (0.807) and Cu - Cr (0.607) (*Table 2*), extracting a group of three principal components. The correlation coefficient of heavy metals in road dust shows a positive correlation of Cu with Fe, Pb, Zn and Cr; Cd with Ni; Fe with Zn and Cr; Pb with Zn; and Zn with Cr (*Table 2*). A positive correlation was observed between Cu-Fe (0.697), Cu-Pb (0.554), Cu-Zn (0.767), Cu-Cr (0.614); Cd-Ni (0.545); Fe-Zn (0.513), Fe-Cr (0.789); Pb-Zn (0.503); and Zn-Cr (0.559). Principal component analysis of heavy metals in PM_10_, PM_2.5_, and road dust samples was performed by extracting a group of three principal components (PC) with a significant eigenvalue of more than 1. The three PC that were extracted accounted for 90.35%, 92.63%, and 88.97% of the data variance in PM_10_, PM_2.5_ and, road dust, as shown in Table 3.

**Table 2:** Correlation Coefficient of Heavy Metals Obtained from Atomic Absorption Spectroscopy Analysis of Contaminants Across Study Locations

PM_10_							

	Fe	Pb	Cd	Ni	Cu	Cr	Zn

Fe	1						
Pb	0.685	1					
Cd	0.477	0.688	1				
Ni	0.567	0.503	0.399	1			
Cu	0.323	0.350	0.733	0.751	1		
Cr	0.070	0.458	0.714	0.111	0.397	1	
Zn	0.498	0.716	0.662	0.026	0.229	0.229	1

PM_2.5_							

	Fe	Pb	Cd	Ni	Cu	Cr	Zn

Fe	1						
Pb	0.668	1					
Cd	0.450	0.193	1				
Ni	0.326	0.417	0.243	1			
Cu	0.443	0.943	0.022	0.411	1		
Cr	0.805	0.715	0.341	0.807	0.607	1	
Zn	0.736	0.651	0.406	0.198	0.423	0.315	1

Road dust							

	Cu	Cd	Fe	Ni	Pb	Zn	Cr

Cu	1						
Cd	0.201	1					
Fe	0.697	0.299	1				
Ni	0.456	0.545	0.346	1			
Pb	0.554	−0.299	0.244	0.254	1		
Zn	0.767	−0.363	0.513	−0.117	0.503	1	
Cr	0.614	0.204	0.789	0.383	−0.01	0.559	1

**Table 3:** Rotated Component Matrix Analysis of Heavy Metals Obtained from Atomic Absorption Spectroscopy Analysis of PM_10_, PM_2.5_, and Road Dust Samples Across Study Locations

PM_10_			

Heavy metal	PC 1	PC 2	PC 3

Fe	0.776	0.491	−0.236
Pb	0.826	0.293	.0295
Cd	0.552	0.366	0.712
Ni	0.170	0.961	0.005
Cu	0.098	0.809	0.468
Cr	0.097	0.059	0.946
Zn	0.894	−0.112	0.264

Eigenvalues	3.796	1.295	1.234
% of variance	54.222	18.497	17.634
Cumulative %	54.222	72.719	90.353

PM_2.5_			

Heavy metal	PC 1	PC 2	PC 3

Fe	0.599	0.208	0.668
Pb	0.939	0.250	0.172
Cd	−0.067	0.180	0.897
Ni	0.156	0.974	0.081
Cu	0.905	0.290	−0.094
Cr	0.538	0.726	0.368
Zn	0.682	−0.382	0.605

Eigenvalues	3.947	1.414	1.123
% of variance	56.387	20.207	16.041
Cumulative %	56.387	76.594	92.635

Road dust			

Heavy metal	PC 1	PC 2	PC 3

Fe	0.746	0.083	0.585
Pb	−0.113	0.86	−0.31
Cd	0.891	−0.008	0.161
Ni	0.253	0.886	0.306
Cu	0.085	−0.034	0.965
Cr	0.696	−0.369	0.429
Zn	0.943	0.25	−0.127

Eigenvalues	3.356	1.807	1.065
% of variance	47.942	25.814	15.215
Cumulative %	47.942	73.755	88.97

### Pollution assessment of heavy metals

The EF, Igeo, CF, PLI, mCd, and RI analysis were used to identify the contamination characteristics of heavy metals. The average EF values for heavy metals in PM_10_ were found to be in a decreasing order of Cd>Pb>Zn>Ni>Cu>Cr. The EF value in PM_2.5_ heavy metals were found to be in the order of Cd>Pb>Zn>Cu>Ni>Cr, and for the road dust, EF values decreased in the order of Pb>Cd>Zn>Cu>Ni>Cr (*Supplemental Material Figure S1*).

Average Igeo index values of PM_10_ were found in the sequence of 1.76, 1.26, 1.17, 1.05, 0.75 and 0.35 for Ni, Pb, Zn, Cu, Cr, and Cd. The Igeo index values across sampling points are shown in Supplemental Material Table S1 to Table S3. All of the Igeo index values were recognized as moderately contaminated or moderately to highly contaminated. On the other hand, the Igeo index value of PM_2.5_ showed an order of Ni (1.81)> Zn (1.33)> Pb (1.28)> Cd (0.36)> Cr (0.30)> Cu (0.28). Negative values signify that the locations were not contaminated with a given heavy metal.

Contamination of PM_10_, PM_2.5,_ and road dust by the heavy metals was calculated with the help of the contamination factor. The CF values showed variation across study locations, indicating variation in contamination levels of the heavy metals. The mCd for heavy metals in PM_10_, PM_2.5,_ and road dust was described for the ranges of mCd values. The mCd values were estimated using CF values of the heavy metals at the representative sites (*Supplemental Material Figure S2 to Figure S4*). The PLI value of heavy metals in PM_10_, PM_2.5,_ and road dust across locations are presented in Supplemental Material Figure S5. Pollution load index values >1 show continuous degradation of the analyzed sampling points.

### Health risk assessment

Cancer and non-cancer health risk values of heavy elements in PM_10_ and PM_2.5_ and road dust via different pathways (inhalation, dermal absorption, and daily intake) are given in Figures Supplemental Material S7–S9.

## Discussion

The PM_10_ and PM_2.5_ concentrations at GVP were 2.9 and 2.8 times higher than the standard values prescribed by the Central Pollution Control Board (CPCB). The National Ambient Air Quality Standards (NAAQS) are 100 μg m^−3^ for PM_10_ and 60 μg m^−3^ for PM_2.5_ for the 24-hour mean (CPCB 2009).^50^ These concentrations were 5.9 times and 8.4 times higher than the World Health Organization (WHO) (air quality guidelines (WHO AQG) value (50 μg m^−3^ for PM_10_ and 25 μg m^−3^ for PM_2.5_; 24-hour mean) (WHO, 2005).^51^ At DBH the concentration of PM_10_ was 2.15 times the NAAQS. Similarly, PM_2.5_ was 2 times higher than the NAAQS value, and these values were 4.3 times and 6 times higher than the WHO AQG for PM_10_ and PM_2.5_ respectively. Higher concentrations of PM generally form haze, which reduces visibility^52,53^ at selected sites in Dhanbad. During the monitoring periods, more than 50% of time, PM_10_ concentrations were above the NAAQS (24-hr average). The concentrations of PM_2.5_ exceeded the 24-h average NAAQS concentration around 75% of the time. Concentrations of PM_10_ and PM_2.5_ values were slightly higher than previously reported values.^23,24^ The DBH site is comparatively clean, as there are fewer sources of pollution and there is a greenbelt around the area. The primary sources of PM at the study locations are dispersed particulates from on-road vehicles such as heavy-duty vehicles, light commercial vehicles, passenger cars, shared auto rickshaws, etc. Particulate matter concentrations are also affected by resuspension of road dust, crustal dust and coal dust derived from coal mining.

### Analysis of heavy metal analysis bound to PM_10_, PM_2.5_, and road dust

Based on the mean concentration of the above locations, concentrations of Fe, Zn, and Pb were found to be the highest among the selected heavy metals in ambient PM and road dust. Levels of these heavy metals in PM_10_ and PM_2.5_ were detected in the order of Fe>Zn>Pb>Cr>Ni>Cu>Cd and Fe>Zn>Pb>Ni>Cu>Cr>Cd, respectively. The overall average concentrations of Fe, Zn, Pb, Cu, Ni, Cr, and Cd in road dust were found to be 38400, 140.84, 63.87, 58.18, 44.12, 41.04 and 0.53 mg kg^−1^, respectively. In comparison to the other heavy metals, the highest concentration was observed for Fe and lowest for Cd. Vehicle exhaust emissions (caused by fuel combustion), non-exhaust emissions (due to tire and brake wear), and coal combustion were the main sources of these heavy metals.^54^ For example, Zn is mainly emitted from wearing of tires^55,56^ and Pb is associated with coal burning.^57^ The concentration of Ni was lower than the concentration of Pb and Zn as less Ni is released from vehicles near the road.^58,59^ Burning of diesel fuel, lubricating oil, and tire wear account for the generation of Cd.^60^ Generally, the concentration of heavy metals was higher in PM_10_ compared to PM_2.5_, and the heavy metal concentration in road dust was found to be lower than in PM_10_ and PM_2.5_, except for Fe. The results of heavy metals in road dust also revealed that the heavy metal (Zn, Cr, Cd, and Ni) concentrations in road dust samples were found to be similar and in the range of PM_10_ and PM_2.5_, indicating that road dust was a sink of heavy metals in PM_10_ and PM_2.5_.

### Source identification of heavy metals in PM_10_ PM_2.5_ and road dust

The positive correlation coefficient of heavy metals in PM_10_ PM_2.5_ and road dust signifies the affinity between the heavy metals.

In PM_10_, component PC1 explained a total data variance of 54.22% with high loadings of Fe, Pb, Cd, and Zn. These heavy metals could be from non-exhaust emissions and re-suspensions of road dust due to vehicular traffic. The presence of Fe indicates that it was emitted by wear and tear of brake pads and vehicle engine parts,^61^ and is a component of the earth's crust.^25^ Lead and Cd are emitted from tire wear and brake linings, coal-burning processes,^62,63^ and Pb is also used in vehicle paint. Zinc is emitted from wear and tear of vulcanized rubber tires, as well as an additive in fuel and through vehicle corrosion.^64,65^ Principal component 2 shows Ni and Cu were responsible for 18.49% of the total data variance with high loadings of Ni and Cu. These heavy metals can be attributed to coal combustion.^66,67^ Heavy metal PC 3 showed a total data variance of 17.63% with high loadings of Cd and Cr. Cadmium and Cr were contributed from automobile exhaust. Chromium is mainly due to engine wear as Cr is one of the metallic components of engines,^68,69^ whereas release of Cd is mainly due to engine wear and the burning of lubricant oil.^70^

In PM_2.5_, from the rotated compound matrix, PC 1 explained 56.38% of the total data variance with high loadings of Fe, Pb, Cu, Cr, and Zn. These heavy metals are contributed from non-exhaust emission and re-suspension of road dust due to vehicular activities. The presence of Fe, Cu and Cr suggest that they may be emitted by wear and tear of brake pads and engine parts.^61^ Copper is a component of the earth's crust.^25^ Sources of Pb include coal combustion, brake wear, fuel, and motor oil combustion and re-suspension of road dust. Dust enriched with Pb exhaust emissions before the phase out of leaded gasoline along with industrial sources have been proposed as the causes of Pb enrichment in roadway dirt.^71^ Zinc is produced from erosion of brake linings and wear and tear of tires from lubricant oil.^55,58,72,73^ Principal component 2 accounted for 20.20% of total data variance with a high loading of Ni and Cr. Previous studies reported the presence of heavy metals such as Ni and Cr due to vehicle emissions.^55,58,72,73^ Nickel is produced from the combustion of fuel and is generated from engine wear.^69,70^ Principal component 3 showed 16.04% of the total data variance with a high loading of Zn, Cd, and Fe. These are attributed to coal combustion.^74,75^

In the principal component analysis of heavy metals in road dust samples, a group of three principal components with an eigenvalue greater than one were extracted *(Table 3)*. Principal component 1 explained 47.942% of the total data variance with high loadings of Cu, Fe, Zn, and Cr, contributed from non-exhaust and re-suspension of road dust, which is generated by vehicle traffic and other nearby activities. Copper, Fe, Zn, and Cr added to the road dust originates from fuel combustion, vehicle emissions such as diesel emissions, tire wear, brake wear, and re-suspension of crustal materials.^76,77^ Principal component 2 showed 25.81% of the total data variance with a high loading of Cd and Ni. These heavy metals are emitted from the burning of lubricant oil. The heavy metals Ni and Cd in PC 2 indicate that they are emitted from vehicles by the burning of lubricant oil and that vehicles can be considered the source of these metals.

Principal component 3 showed 15.21% of the total data variance with a high loading of Cu and Pb. Copper and Cd are heavy metals released from coal-burning.^78,79^

Principal component analysis of PM_10_, PM_2.5_, and road dust showed that vehicles are the primary source of the selected heavy metals. Local activities such as coal combustion and ground level resuspension of dust are other sources of heavy metals in PM_10_, PM_2.5_, and road dust. These heavy metals may come from non-exhaust sources and vehicle exhaust. Non-exhaust sources include tire wear, brake wear, and erosion of vehicle parts. Vehicle exhaust emissions include engine wear and fuel combustion, and other sources include coal combustion and resuspension of crustal materials and dust, which are enriched with heavy metals in the road dust. Resuspension of road dust due to tire abrasion depends on metrology, condition of the road surface and the volume of vehicles on the road.^71^ Burning of coal in stoves is actively practiced in local shops near the roadways and is a source of emission of PM into the atmosphere.^80^

### Pollution assessment of heavy metals

The EF values of the heavy metals in PM_10_ PM_2.5_ and road dust at the selected location is depicted in Supplemental Material Figure S1. The EF values of heavy metals were observed in three categories: slightly enriched, moderately enriched, and highly enriched. Heavy metals in road dust were mainly from crustal and anthropogenic sources. In PM_10_, and PM_2.5,_ the EF value indicated the abundance of anthropogenic sources. Cadmium, Pb, and Zn were found to be highly enriched, whereas Cr, Cu and Ni were moderately enriched in PM_10_ and PM_2.5._ The overall values showed that anthropogenic activities such as coal burning, vehicular exhaust emissions, and non-exhaust emissions (tire and brake wear) and re-suspension mainly contributed to PM_10_, PM_2.5,_ and road dust.

The Igeo index value across study locations ranged between uncontaminated, slightly contaminated, and moderately contaminated. The Igeo index value of road dust across study sites is presented in Supplemental Material Table S1 to *S3*. I_geo_ index values were considered to be uncontaminated to slightly contaminated and moderately to highly contaminated. Only the I_geo_ index value for Zn at GVP was recognized as highly contaminated.

The CF values of heavy metals present in PM_10_, PM_2.5,_ and road dust at selected sites are shown in Supplemental Material Figure S2 to S4. Contamination levels were classified as: CF < 1 = low contamination (indicating low sediment contamination of the heavy metals); 1 ≤ CF < 3 = moderate contamination; 3 ≤ CF < 6 = considerable contamination, and CF ≥ 6 = very high contamination.^77^ At the selected locations, CF values varied from moderately contaminated, moderately to strongly contaminated, strongly contaminated, and very strongly contaminated.

The value of mCd was observed between a moderate degree of contamination and a high degree of contamination in PM_10_, PM_2.5,_ and road dust at selected sites. The mCd value of the heavy metals in PM_2.5_ at SCH, CMR, IGT, GSC, and GVP showed a high degree of contamination, while at DBH the value showed a moderate degree of contamination. Based on the mCd values for heavy metals in PM_10_, the mCd signified a high degree of contamination at all locations except for DBH. At DBH, a moderate degree of contamination was found. In road dust, the observed value of mCd revealed a high degree of contamination at SCH, CMR, IGT, and GVP, while it indicated a moderate degree of contamination at GSC and DBH. The mCD values at selected locations are given in Supplemental Material Figures S2 to S4.

The PLI value for PM_10_ was highest at CMR with a PLI value of 4.37, and the PLI value for PM_2.5_ at CMR was highest with a value of 3.74. For road dust, the PLI value was observed to be the highest at GVP. The PLI values of road dust at SCH, IGT, and GVP were greater than for PM_10_, and PM_2.5_ which signifies that the road dust was highly contaminated. Different risk index values of heavy metals in PM_10_, PM_2.5,_ and road dust are presented in Supplemental Material Figure S7. The RI value estimated in PM_10_ indicates that no ecological risk was found at SCH, CMR, IGT, GSC, DBH, and GVP (RI values <150). Based on RI values in PM_2.5_, no ecological risk was observed at SCH, CMR, IGT, GSC and DBH (RI values <150), but the RI values at GVB were >150 which signifies that these locations had moderate ecological risk. The RI values in the road dust indicate that moderate ecological risk was found at IGT, GSC, and DBH (RI value <150), whereas IR values at SCH, CMR, and GVP were found to be greater than 150, which indicates that these locations had moderate ecological risk.

### Health risk assessment

The hazard quotient (HQ) values of all the heavy metals via three different pathways in the contaminants were highest through ingestion, followed by dermal and inhalation for both children and adults. These results indicate that ingestion and dermal contact were the main routes of health risk exposure for the considered heavy metals for children and adults. The hazard index (HI) is the sum of all the HQs from different routes. The HI values of the heavy metals in PM_10_ and PM_2.5_ were less than 1 for Cu, Cd, Ni, Pb, Zn, and Cr for both children and adults across study locations. It was also observed that adults and children were exposed to the minimum risk at BKG in comparison to SCH, CMR, IGT, GSC, DBH, and GVP. The HI value of heavy metals in road dust at all the locations were lower than 1. An HI value lower than 1 indicates that the health risk of the concerned heavy metals in the contaminant presented no non-carcinogenic adverse risks to the health of children and adults. The HI values of the heavy metals were found in order of PM_2.5_>PM_10_>road dust.

The carcinogenic risks posed by Cu, Cd, Ni, Pb, Zn, and Cr in PM_10_, PM_2.5_, and road dust for children and adults at all the locations were lower than the acceptable tolerance value 1×10^6^ – 1×10^−4^,^81^ indicating that the heavy metals present no carcinogenic risks to the health of children and adults across study locations.

## Conclusions

In the present study, PM_10_, PM_2.5_, and road dust samples for the selected road network of Dhanbad were collected, and their mass concentration, heavy metal concentration, source apportionment, and ecological and health risks were investigated. The average values of PM_10_ (229.54±118.40 μg m^−3^) and PM_2.5_ (129.73 ±61.74 μg m^−3^) were 2.29 and 2.16 times higher than the standard value, indicating serious PM contamination in the study area. The particulate-bound heavy metals in contaminants showed high values for Cd, Pb, and Zn. The principal component analysis results indicated that vehicular activities are the most significant contributors to heavy metals. The contamination level study explains the difference in contamination level due to variation in the heavy metals present across study locations. The RI values indicate that most heavy metals only presented moderate and low risks in all the samples. Based on health risk assessment, the non-carcinogenic risks posed by Cu, Cd, Ni, Pb, Zn, and Cr were less than 1. Children were more greatly affected than adults *(Supplemental Material Figures S7–S9).* The carcinogenic risk posed to children and adults by the heavy metals was below the tolerance value of 1 × 10^−6^–1 ×10^−4^ at the selected locations. Health risks posed by PM_10_, PM_2.5,_ and road dust for adults and children can be minimized by reducing time spent outdoors and wearing a mask.^53^ The adverse health effects of PM and road dust exposure can be reduced by spreading awareness among individuals. Widening of roads can reduce the movement of vehicles on unpaved portions of the roadway. Paved shoulders on both sides of the road may also reduce the resuspension of dust caused by vehicular movement. Proper road maintenance and adequate vehicle maintenance may also be helpful to reduce PM emissions.

The limitations of the present study are that samples were collected in the winter season only, and road dust samples were not size segregated. Size segregation of PM_10_, PM_2.5_, and road dust may give more specific results. In addition to heavy metal analysis, other components of PM, such as organic carbon, elemental carbon, and polyaromatic hydrocarbons should be included in future studies.
