# Supplementary material for: Assessment of Pollution and Health Risks of Heavy Metals in Particulate Matter and Road Dust Along the Road Network of Dhanbad, India
Source: J Health Pollut. 2021 Mar 2;11(29):210305. doi: 10.5696/2156-9614-11.29.210305 (PMC8009640; doi:10.5696/2156-9614-11.29.210305)
Supplement: Supplementary file 1 [file Jain_Supplemental_Material.docx]

**Supplemental Material**

**Text S1. Sample preparation for analysis of heavy metals bound to PM_10_ and PM_2.5_**

The heavy metals present in PM and blank samples were determined by acid digestion. For extraction of heavy metals bound to PM_10_ and PM_2.5_ from the samples, one-fourth of the EPM 2000 filter papers and whole PTFE filter papers were digested in a Teflon vessel of a microwave digester (Ethos One, Italy). Twenty (20) ml of HNO₃ (65% GR grade, Merck) and 2 ml HClO_4_ (70% GR grade, Merck) acids were used in the digestion. The microwave digester was operated at 210°C for 10 minutes and incubated at 210°C for 15 minutes. After digestion, the solution was filtered using Whatman 42 filter paper. The filtrates of PM_10_ and PM_2.5_ were diluted to 50 ml and 25 ml, respectively, with Milli-Q water and kept in Tarson bottles in a refrigerator until analysis.

**Text S2. Road dust sample preparation for heavy metal analysis**

An aliquot sample of 0.5 grams of oven-dried and sieved road dust from selected locations and a background site were weighed and placed in a Teflon vessel with 20 mL of HCL and HNO_3_ in the ratio of 3:1.**^1^** These samples were kept overnight for better mixing then the Teflon vessels were heated until it became a transparent solution. These transparent samples were passed through Whatman no. 42 filter paper and diluted to 20 mL using distilled water. These samples were stored in a Tarson bottle in the refrigerator under 4°C until AAS analysis.

The concentrations of seven (Fe, Pb, Cd, Ni, Cu, Cr, and Zn) heavy metals in PM and road dust were determined using AAS (GBC Avanta PM, Australia). The detection limit of the instrument was 0.005, 0.01, 0.0004, 0.009, 0.001, 0.003, 0.0005 ppm for Fe, Pb, Cd, Ni, Cu, Cr, and Zn, respectively. The accuracy of the analytical procedure adopted for AAS analysis was repeatedly verified by running standard solutions after every ten samples. All the samples were analyzed in triplicates.

**Text S3. Contamination assessment in PM10, PM2.5 and road dust samples**

1. *Enrichment Factor*

The enrichment factor was used to differentiate the elements of anthropogenic origin from natural sources within the atmospheric PM and road dust. **^2–4^** Enrichment factor values for elements were calculated for the investigated elements in samples. The equation used to calculate the EF values for elements in samples is Equation 1:

 Equation 1

where C_n_ is the average concentration of the element considered in the studied samples or the earth’s crust, and C_Fe_ is the average concentration of the reference element Fe in the studied samples or the earth’s crust. Iron is used as a reference element. An EF <10 specifies an abundance of crustal sources for that element; an EF > 10 suggests that the element has an abundance of anthropogenic sources. An EF of 10 < EF < 100 indicates that elements are moderately enriched, while an EF > 100 indicates that elements are anomalously enriched by anthropogenic activities.**^5,6^**

1. *Geo-accumulation index*

The geo-accumulation index is a parameter used to relate the heavy metal concentrations with background values of atmospheric PM and road dust. The Igeo was calculated by using Equation 2:**^2, 7^**

 Equation 2

where Cn is the average concentration of heavy metals in the samples and Bn is the concentration of heavy metals in the background sample. A factor of 1.5 was used to offset the effect of possible fluctuations in background values.**^8^** According to Muller,**^9^** the value of Igeo, the degree of heavy metal contamination, can be classified into seven groups, as presented in Table 2.^10^

Table 2: Classification of Contamination Level According to Igeo Range

| Value | Contamination level |
| --- | --- |
| Igeo< 0 | Uncontaminated |
| 0 - 1 | Uncontaminated to moderately contaminated |
| 1 - 2 | Moderately contaminated |
| 2 - 3 | Moderately to heavily contaminated |
| 3 - 4 | Heavily contaminated |
| 4 - 5 | Heavily to extremely contaminated |
| Igeo> 5 | Extremely contaminated |

1. *Contamination factor*

The contribution of heavy metals in the samples due to anthropogenic activities can be predicted from their enhancement compared to background levels. Different pollution indicators such as contamination factor (CF), contamination degree (Cd), modified degree of contamination (mCd), risk index (RI), and pollution load index (PLI) can be used to provide a comparative study of different sampling sites. Håkanson classification (1980),**^13^** the CF, and Cd were used for the calculation of the contamination status of air quality and dust as environmental pollution indicators in the present study. The total degree of contamination at a given location was calculated by the modified degree of contamination. **^11^** The contamination factor, Cd, and mCd for each heavy metal in the samples were determined by Equations ‌3-‌5:

 Equation 3

where Cb refers to the concentration of targeted heavy metals in the background samples, and Cs is the concentration of heavy metals found in the samples. According to Håkanson (1980): Cf < 1 indicates low contamination; Cf 1-3, moderate contamination; Cf 3-6, considerable contamination; and Cf > 6 indicates very high contamination (12).

 Equation 4

According to the Håkanson classification: Cd < 6 shows a low degree of contamination; Cd 6-12 is a moderate degree of contamination; Cd 12- 24 is a considerable degree of contamination; and Cd > 24 is a high degree of contamination indicating serious anthropogenic pollution.

 Equation 5

In Equation 5, n = number of analyzed heavy metals, i = i^th^ heavy metals, and CF = contamination factor. For the classification and description of the modified degree of contamination (mCd) in the sample, the subsequent gradations are proposed: mCd < 1.5 indicates nil to a very low degree of contamination, ≤1.5 mCd <2 indicates a low degree of contamination, mCd 2 ≤ mCd <4 indicates a moderate degree of contamination, mCd 4 ≤ mCd< 8 indicates a high degree of contamination, 8 ≤ mCd <16 indicates a very high degree of contamination, 16 ≤ mCd <32 indicates an extremely high degree of contamination, and mCd ≥ 32 indicates an ultra-high degree of contamination.

The PLI provides knowledge of the collective pollution load from the total toxic metals at the site**^12^** and was used to assess the air quality and road dust quality of the sampling sites. The PLI for each site was calculated from the CF formula given in Equation 6:

 Equation 6

where CF is the contamination factor for a single heavy metal and PLI<1 signifies no pollution, PLI = 1 indicates only baseline levels of contaminations, and PLI>1 reveals deterioration of site quality.**^12^**

The potential ecological risk index (RI) was evaluated to assess the ecological risk of contamination of multi-metals in the samples. The RI was proposed by Håkanson (1980) and is commonly used by ecologists or environmentalists. **^13–17^** The RI and potential ecological risk coefficient ($E_{r}^{i}$) are calculated by Equations 7 - 9:

$\text{C}_{\text{f}}^{\text{i}}\text{=C}_{\text{s}}^{\text{i}}\text{/C}_{\text{n}}^{\text{i}}\text{ }$ Equation 7

$\text{E}_{\text{r}}^{\text{i}}\text{=T}_{\text{r}}^{\text{i}}\text{×C}_{\text{f}}^{\text{i}}\text{ }$ Equation 8

$\text{RI=}\sum_{\text{i=1}}^{\text{n}} \text{E}_{\text{r}}^{\text{i}}\text{ }$ Equation 9

where $C_{f}^{i}$ is the pollution coefficient of a single element of ‘I’; $C_{s}^{i}$ the measured level of sedimentary heavy metal; $C_{n}^{i}$ the background level of sedimentary heavy metal; and $T_{r}^{i}$ the toxic response factor (i.e., Cd = 30, Pb = Cu = Ni = 5; Cr = 2; and Zn = 1). According to Håkanson,**^13^** $E_{r}^{i}$ <40 indicates low ecological risk, 40 ≤$E_{r}^{i}$ ≤80 indicates moderate ecological risk, 80 ≤$E_{r}^{i}$ ≤160 indicates considerable ecological risk, 160 ≤ $E_{r}^{i}$ ≤320 indicates high ecological risk, and 320 ≤$E_{r}^{i}$ indicates serious ecological risk (ii). An RI ≤ 150 indicates a low potential ecological risk, 150 ≤ RI ≤ 300 indicates a moderate potential ecological risk, 300 ≤ RI ≤ 600 indicates a considerable ecological risk, and 600 ≤ RI indicates a very high ecological risk.**^18^**

**Text S4. Human health risk assessment**

The average daily dose (ADD) of heavy metals via three different pathways were calculated by the following equations (10)-(12): **^19, 20^**

 Equation 10

 Equation 11

 Equation 12

The details of the variables used in Equations (10) – (12) are given in Table 1 (of this supplemental material).

After the ADD estimation for ingestion, inhalation, and dermal exposure pathways, the non-carcinogenic risk by the heavy metals in PM and road dust was quantified by the hazard quotient (HQ). The HQ is calculated by Equations (13) **^19, 21^** and the carcinogenic risks (CR) posed by heavy metals in PM and road dust via inhalation were calculated by Equation (15):

Equation 13

Equation 14

$$HI = \sum HQ$$

Equation 15

where ADD is the average daily dose for the exposure of heavy metal via ingestion, inhalation, and dermal pathways. The RfD (mg kg^-1^day^-1^) is the reference dose. The HI is the summation of multiple exposure pathways of HQ. There is concern about the potential for non-carcinogenic effects if the exposure level of a heavy metal exceeds the limit HI >1. However, if HI≤1, then the non-carcinogenic risk posed is insignificant.**^22^** SF_inh_ is the cancer slope factor for the inhalation pathway of the heavy metals in the contaminant. For regulatory purposes, the CR value is considered as an acceptable or tolerable risk only in the range of 1 × 10^−6^–1 ×10^-4^ .^23^ The values of RfD and SFinh used in the present study are given in Table 2.

Table 1: Recommended Values Used in Equations for Exposure Dose of Heavy Metals in Particulate Matter and Road Dust

| Parameter | Definition (unit) | Value | | Reference |
| --- | --- | --- | --- | --- |
|  |  | Children | Adult |  |
| C | Average concentration of the heavy metal in road dust (mg kg^-1^) |  |  | This study |
| Ring | ingestion rate (mg day^-1^) | 200 | 100 | **^21^** |
| EF | exposure frequency (days year^-1^) | 180 | 180 | **^24^** |
| ED | exposure duration (years) | 6 | 24 | **^25^** |
| BW | average body weight (kg) | 15 | 70 | **^25^** |
| AT | average time (days) |  |  | **^25^** |
|  | AT for non- carcinogenic heavy metals (days^-1^) | ED×365 | ED×365 |  |
|  | AT for carcinogenic heavy metals (days^-1^) | 365×70 | 365×70 |  |
| CF | conversion factor kg mg^-1^ | 1.00E-06 | 1.00E-06 | **^25^** |
| Rinh | inhalation rate (m^3^ day^-1^) | 7.6 | 20 | **^25^** |
| PEF | particle emission factor m^3^ kg^-1^ | 1.36E+09 | 1.36E+09 | **^25^** |
| SA | surface area exposed to dust cm^2^ | 2800 | 5700 | **^25^** |
| AF | skin adherence factor (mg cm^2^ d^-1^) | 0.2 | 0.7 | **^25^** |
| ABS | dermal absorption factor | 0.001 | 0.001 | **^25^** |

Table 2: Reference Dose (mg kg^-1^day^-1^) and Slope Factor Values Used in Equations to Evaluate the Risk of Heavy Metals

| Heavy elements | Cd | Cr | Cu | Ni | Pb | Zn |
| --- | --- | --- | --- | --- | --- | --- |
| RfDing | 1.00E-03 | 3.00E-03 | 4.00E-02 | 2.00E-02 | 3.50E-03 | 3.00E-01 |
| RfDinh | 1.00E-03 | 6.00E-05 | 1.20E-02 | 5.40E-03 | 3.52E-03 | 3.01E-01 |
| RfDderm | 1.00E-05 | 2.86E-05 | 4.02E-02 | 2.06E-02 | 5.25E-04 | 6.00E-02 |
| SF | 6.30E+00 | 4.20E+01 |  | 8.40E-01 |  |  |

RfD_ing_ = Ingestion reference dose

RfD_inh_ = Inhalation reference dose

RfD_erm_ = Dermal reference dose

SF = Slope factor


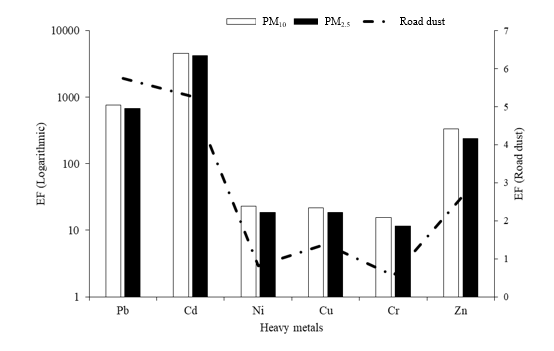


Figure S1. Enrichment factor for heavy metals in PM_10_ PM_2.5_ and road dust calculated with respect to the

earth's crustal composition.


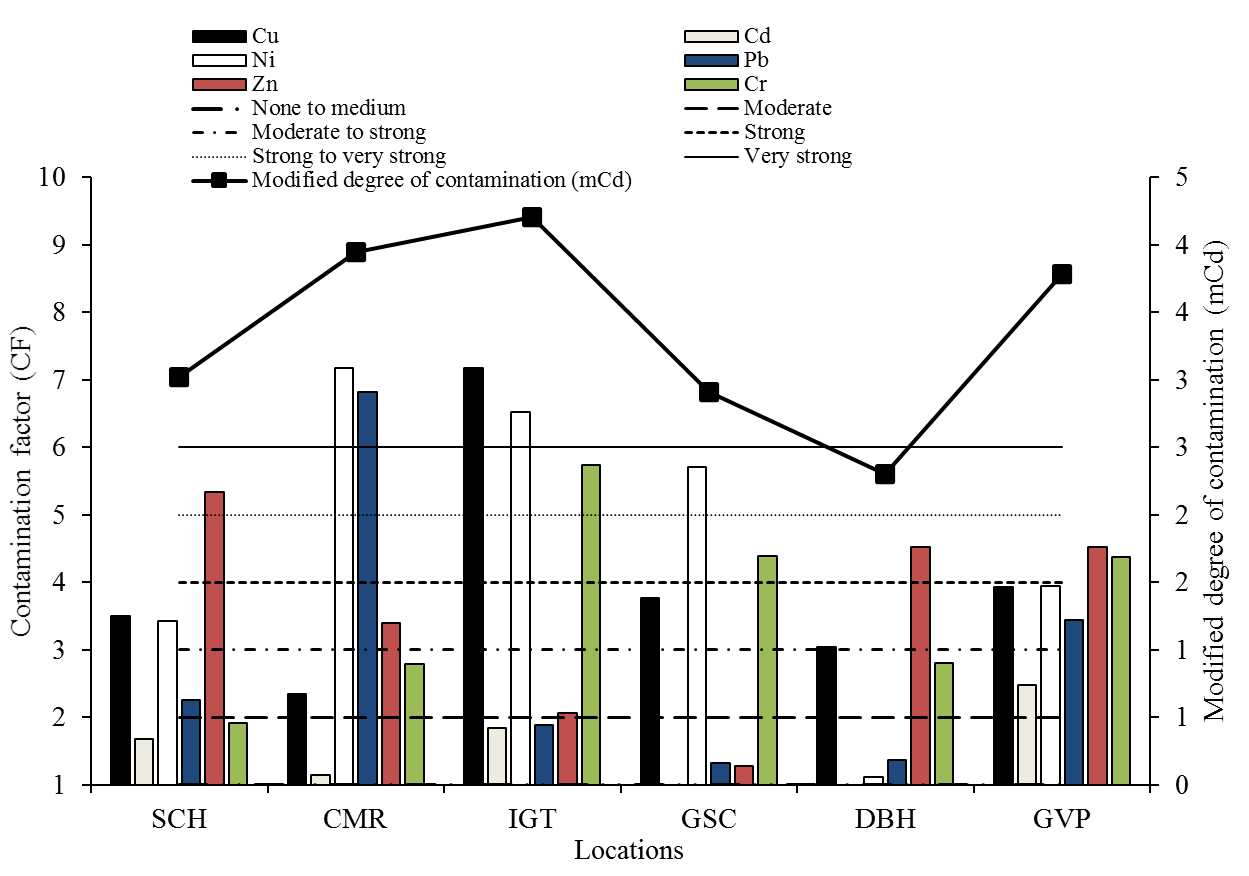


Figure S2. Contamination factor and degree of contamination of PM_10_ by heavy metals in selected areas.


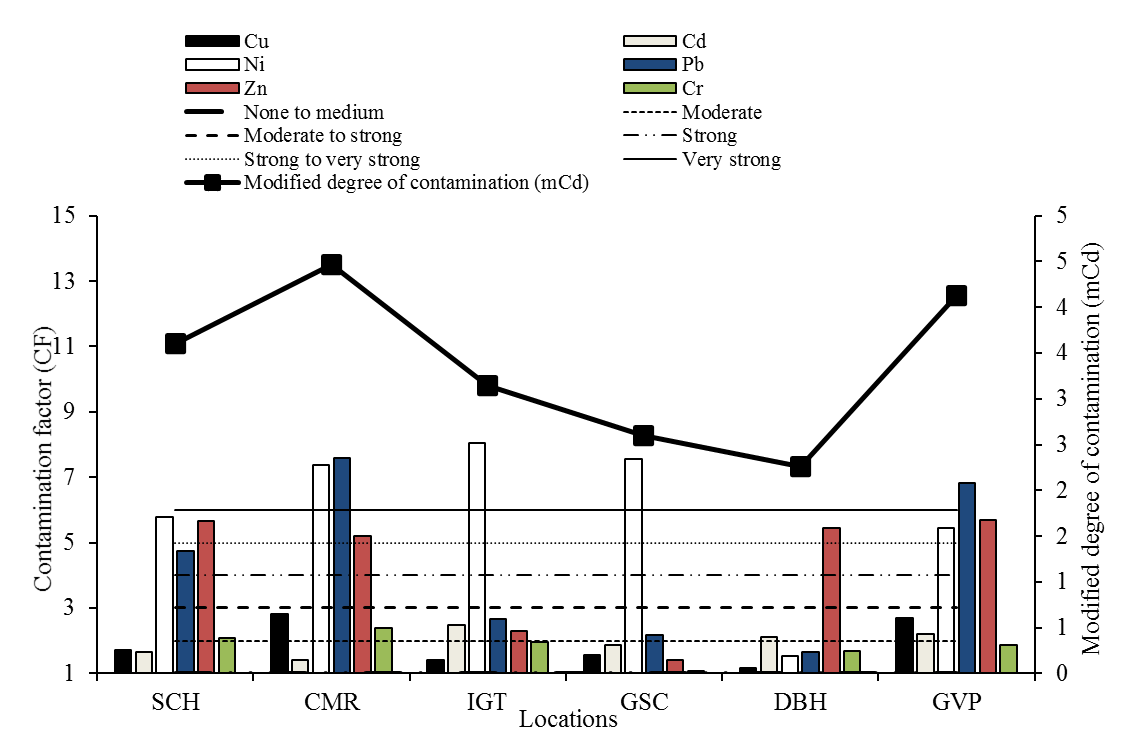


Figure S3. Contamination factor and degree of contamination of PM_2.5_ by metals in selected areas.


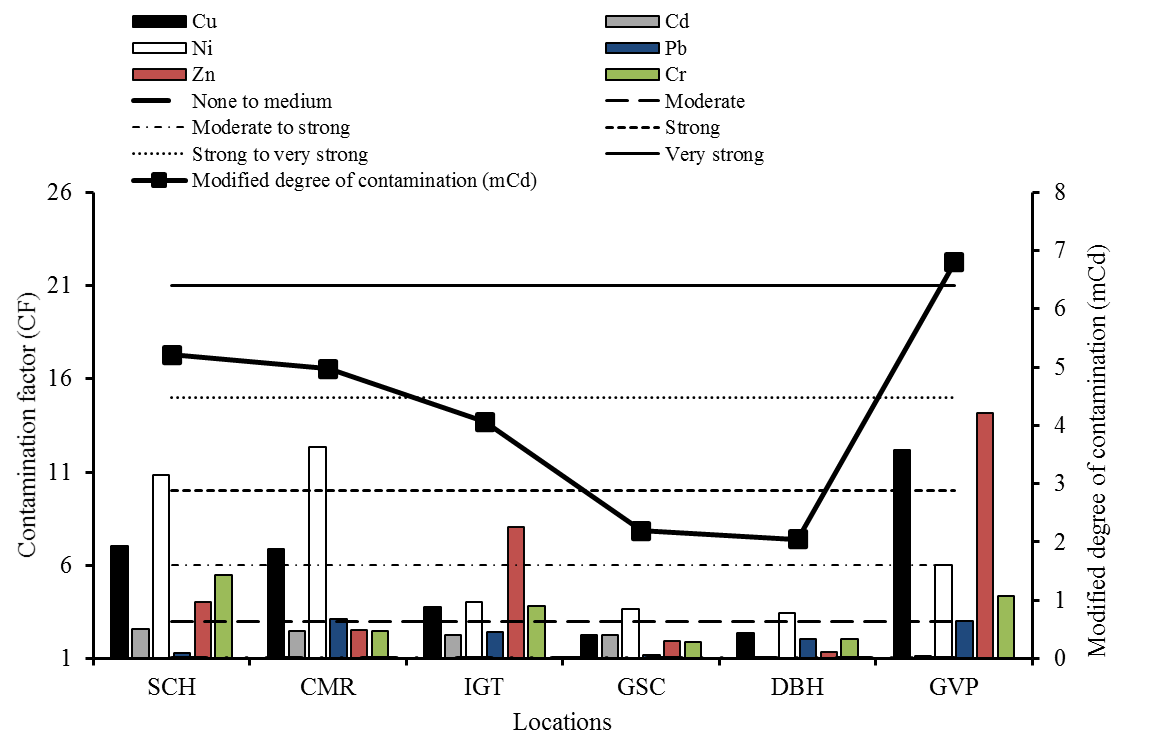


Figure S4. Contamination factor and degree of contamination of road dust by metals in selected areas.

Table S1 : I_geo_ Values for Heavy Metals in PM_10_ Across Study Locations

| Igeo PM_10_ | | | | | | |
| --- | --- | --- | --- | --- | --- | --- |
| Locations | Cu | Cd | Ni | Pb | Zn | Cr |
| SCH | 0.88 | 0.20 | 1.69 | 1.70 | 1.61 | 0.68 |
| CMR | 1.74 | -0.16 | 2.45 | 2.29 | 1.77 | 1.17 |
| IGT | 0.62 | 0.73 | 2.41 | 0.85 | 0.35 | 0.72 |
| GSC | 1.21 | 0.24 | 1.91 | 0.49 | -0.22 | 1.06 |
| DBH | 0.23 | 0.49 | 0.46 | -0.06 | 1.74 | 0.41 |
| GVP | 1.60 | 0.57 | 1.65 | 2.27 | 1.77 | 0.45 |
| Average | 1.05 | 0.35 | 1.76 | 1.26 | 1.17 | 0.75 |

Table S2: I_geo_ values for Heavy Metals in PM_2.5_ Across Study Locations

| Igeo PM_2.5_ | | | | | | |
| --- | --- | --- | --- | --- | --- | --- |
| Locations | Cu | Cd | Ni | Pb | Zn | Cr |
| SCH | 0.19 | 0.13 | 1.95 | 1.66 | 1.91 | 0.47 |
| CMR | 0.90 | -0.10 | 2.30 | 2.34 | 1.79 | 0.68 |
| IGT | -0.10 | 0.73 | 2.42 | 0.83 | 0.62 | 0.62 |
| GSC | 0.04 | 0.31 | 2.33 | 0.54 | -0.11 | -0.50 |
| DBH | -0.21 | 0.50 | 0.02 | 0.14 | 1.86 | 0.16 |
| GVB | 0.85 | 0.56 | 1.86 | 2.19 | 1.92 | 0.39 |
| Average | 0.28 | 0.36 | 1.81 | 1.28 | 1.33 | 0.30 |

Table S3: I_geo_ values for Heavy Metals in Road Dust Across Study Locations

| Igeo Road dust | | | | | | |
| --- | --- | --- | --- | --- | --- | --- |
| Locations | Cu | Cd | Ni | Pb | Zn | Cr |
| SCH | 1.00 | 0.01 | 1.61 | 1.80 | 2.14 | 1.41 |
| CMR | 2.01 | -0.04 | 2.70 | 2.79 | 2.10 | 2.14 |
| IGT | 0.83 | 0.66 | 2.76 | 0.62 | 0.88 | -0.09 |
| GSC | 1.47 | 0.42 | 2.50 | 0.35 | -0.01 | 1.44 |
| DBH | 0.37 | 0.67 | 0.27 | -0.09 | 1.82 | -0.07 |
| GVB | 2.62 | 0.52 | 2.36 | 2.73 | 2.32 | 0.80 |
| Average | 1.38 | 0.37 | 2.03 | 1.37 | 1.54 | 0.94 |


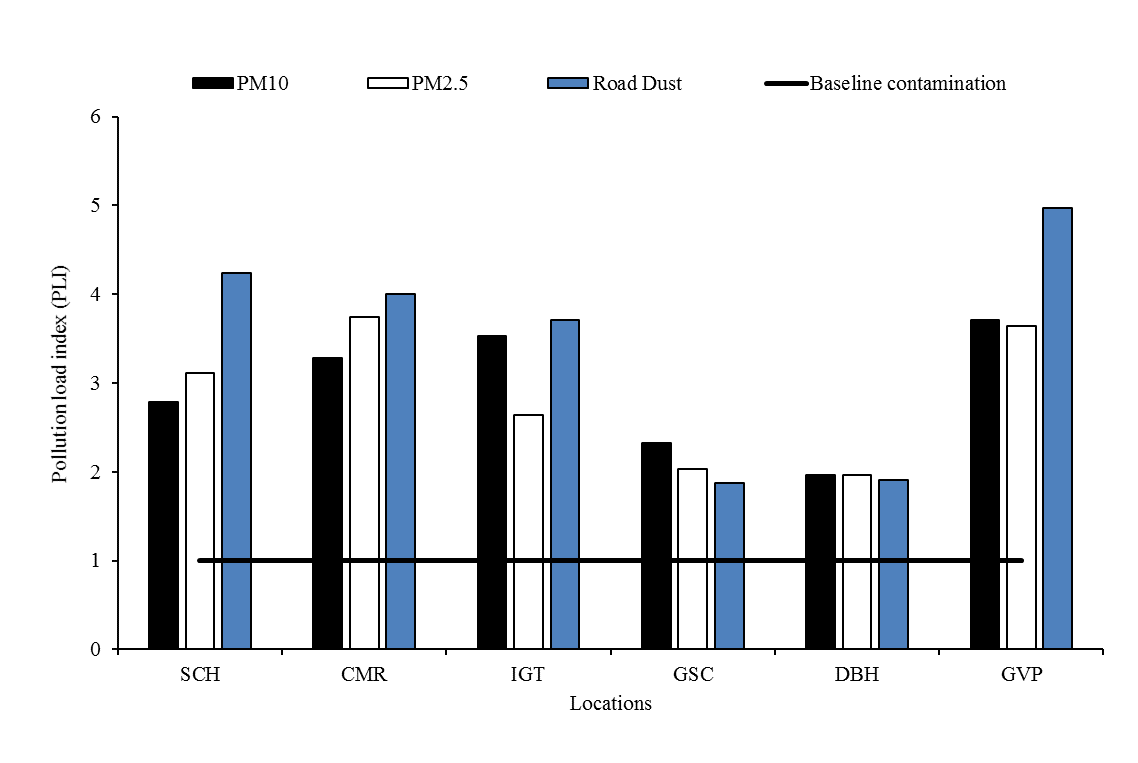


Figure S5. Pollution load index of heavy metals in PM_10_ PM_2.5_ and road dust of sampling sites


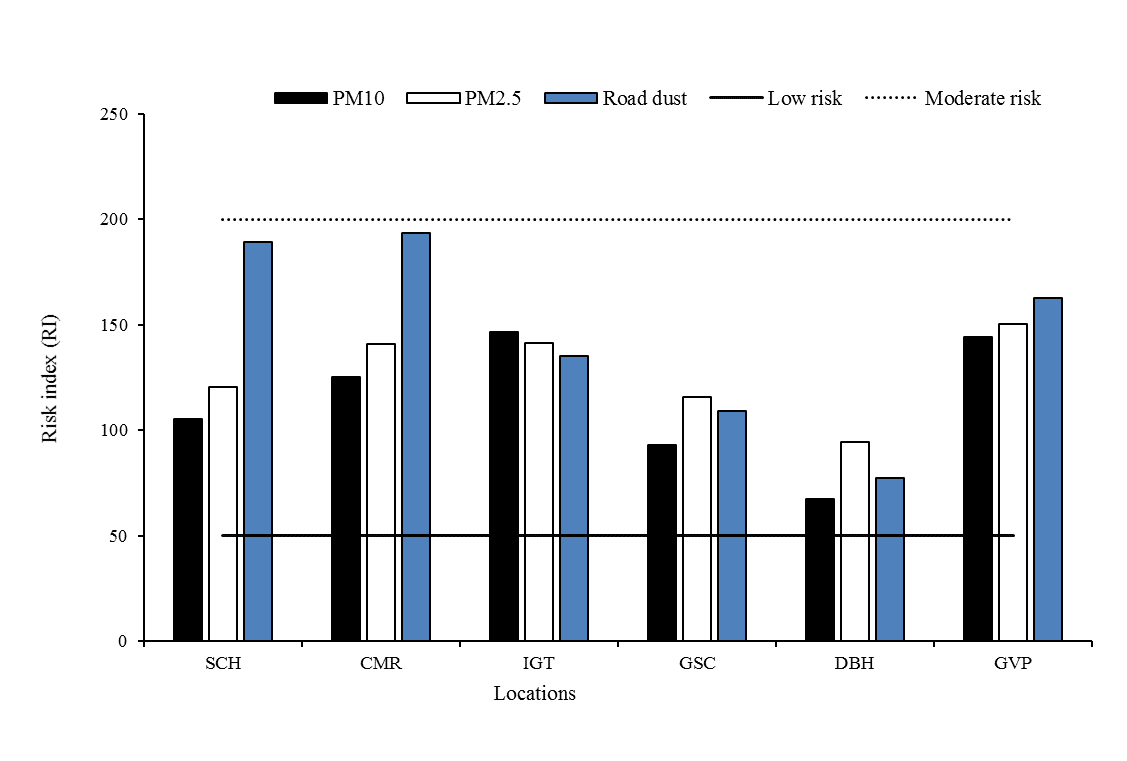


Figure S6. Risk index variation of heavy metals in PM_10_, PM_2.5_, and road dust at selected sites.

| 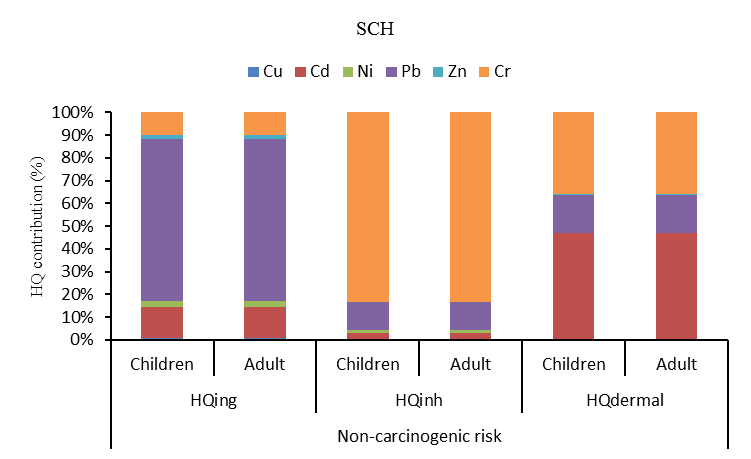 | 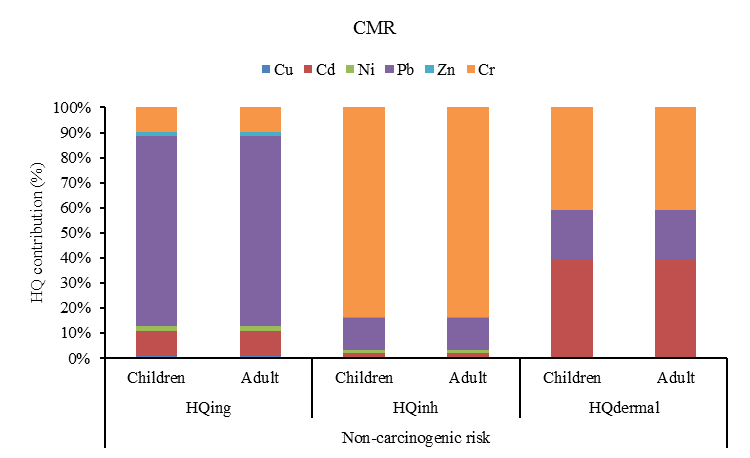 |
| --- | --- |
| 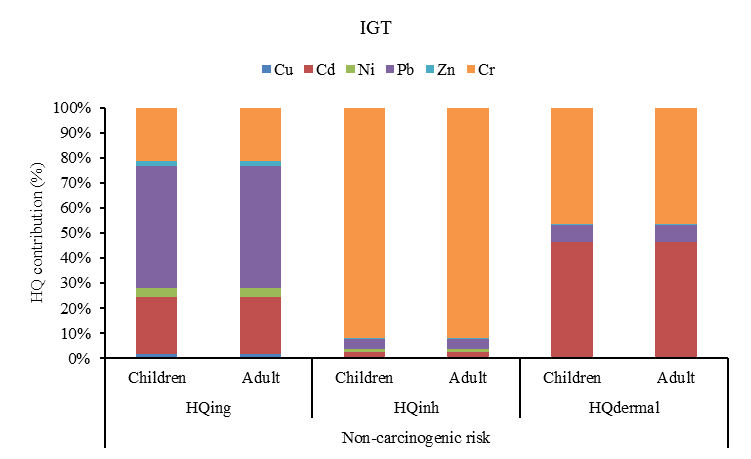 | 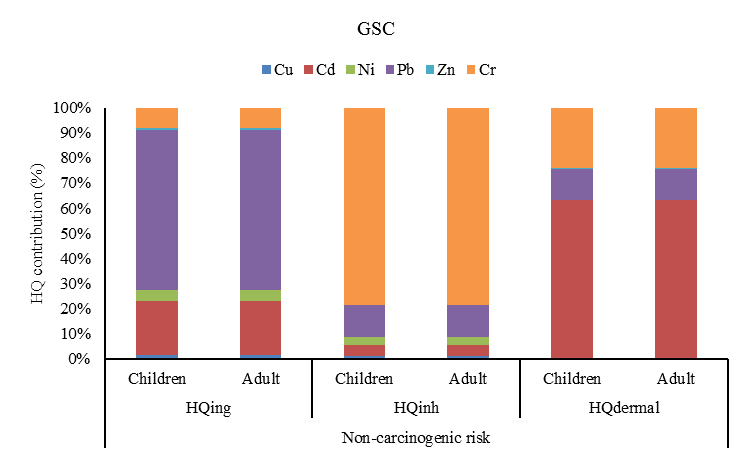 |
| 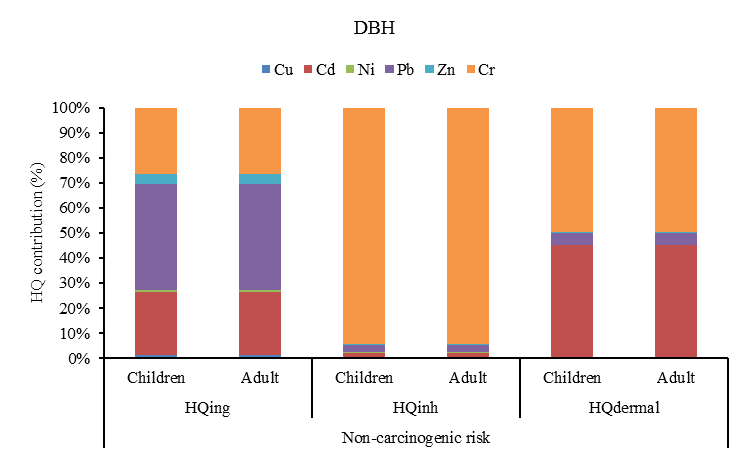 | 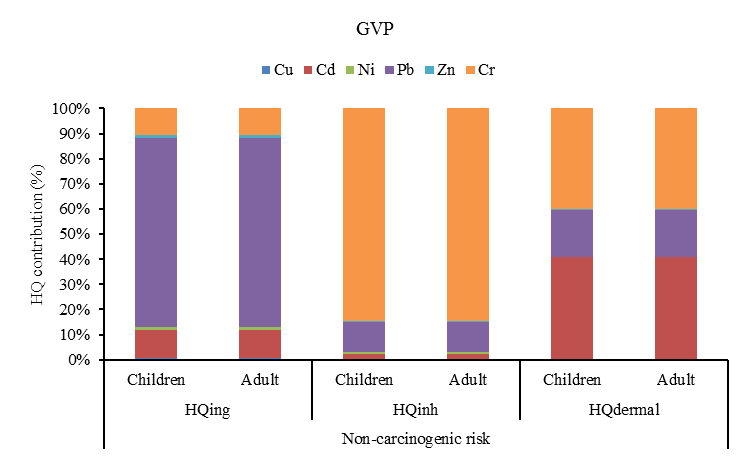 |


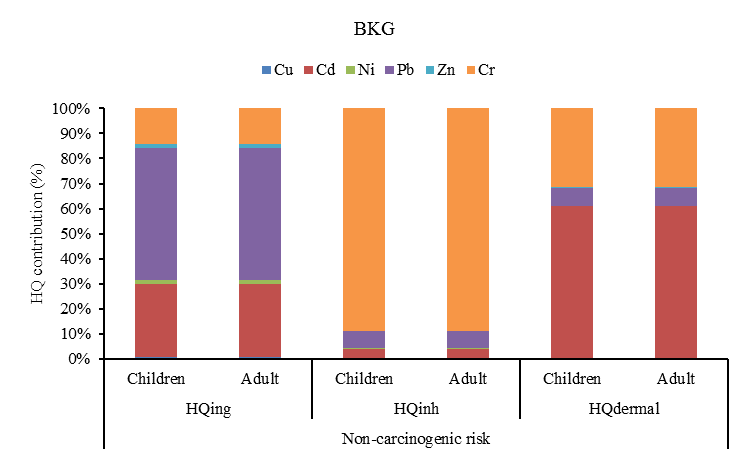


Figure S7: Hazard quotients for three pathways of exposure and carcinogenic risks posed by heavy metals in PM_10_ in children and adults.

| 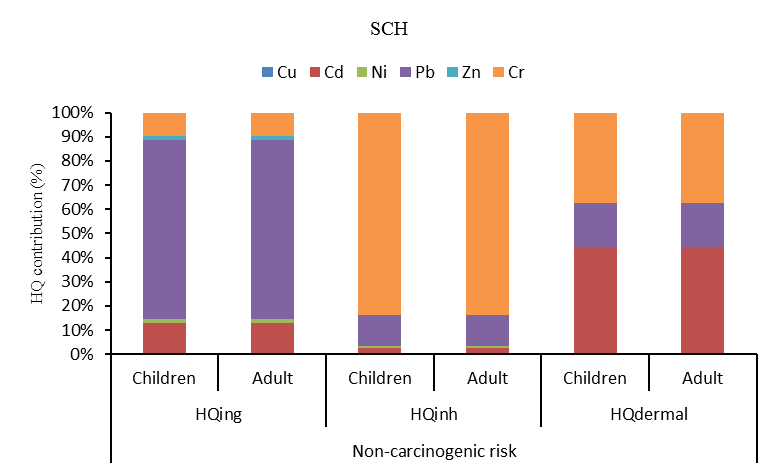 | 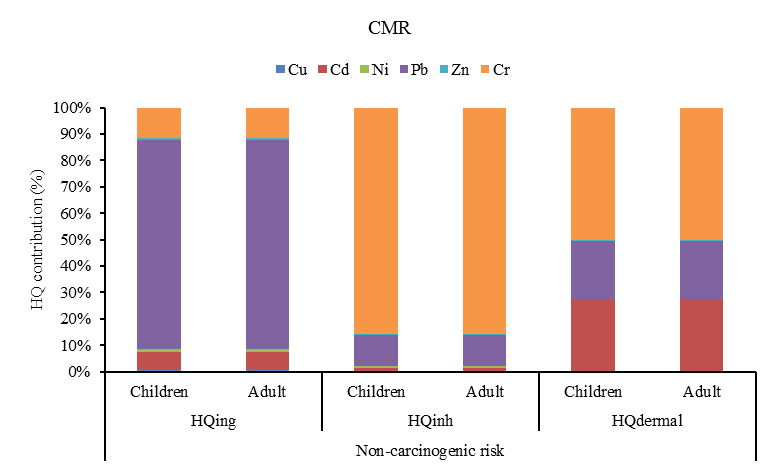 |
| --- | --- |
| 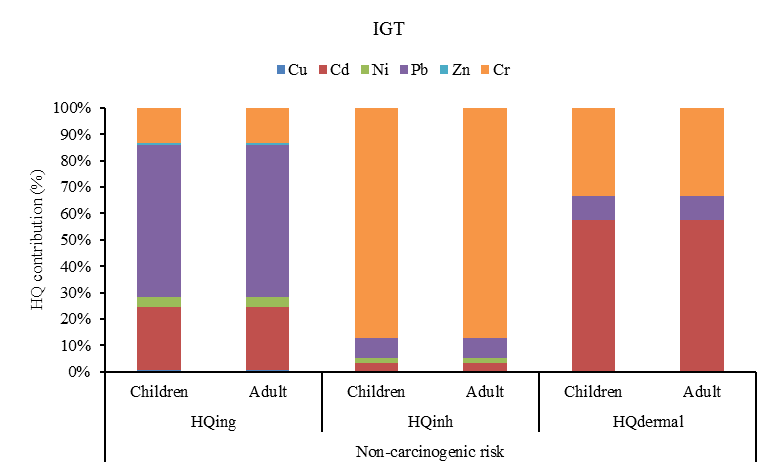 | 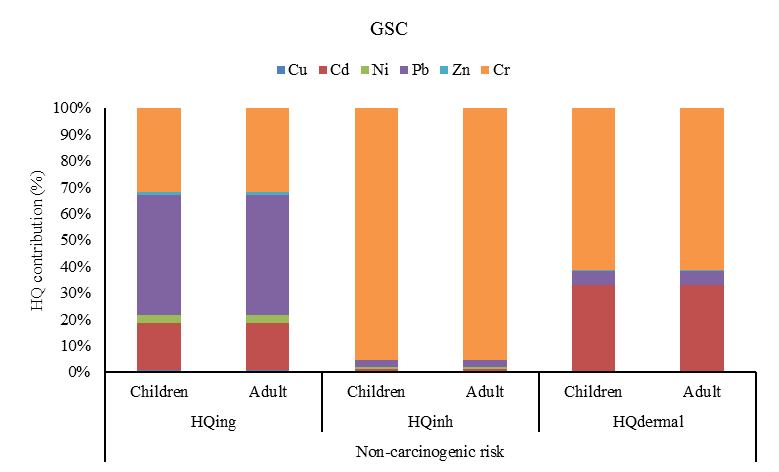 |
| 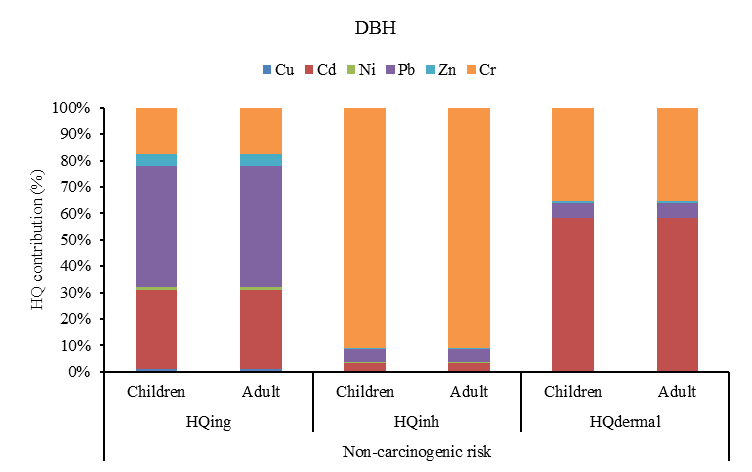 | 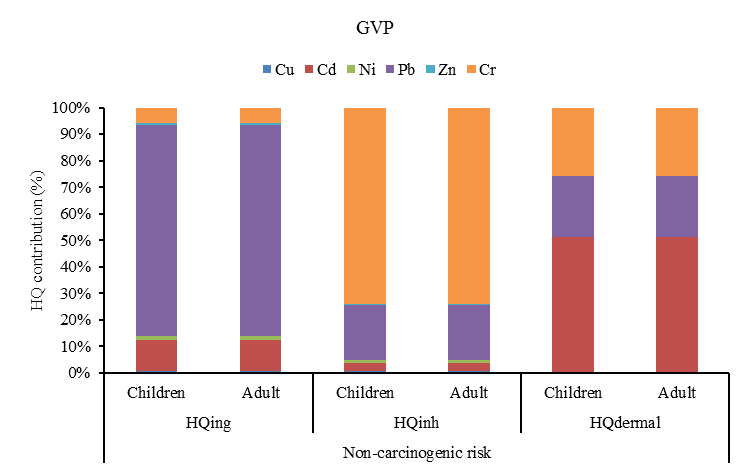 |


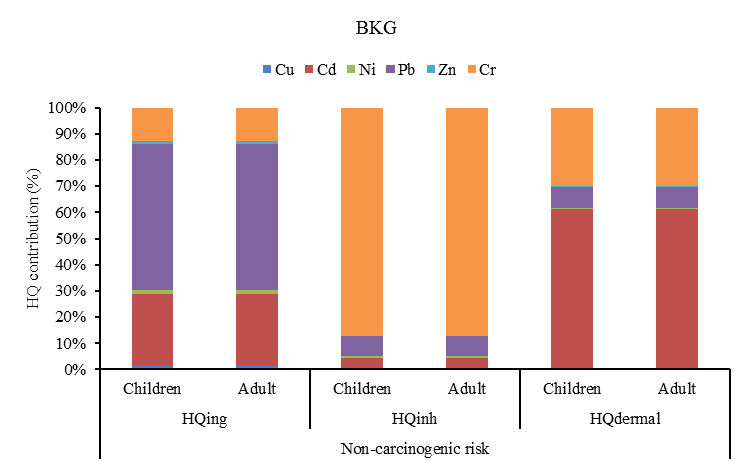


Figure S8: Hazard quotients for three pathways of exposure and carcinogenic risks posed by heavy metals in PM_2.5_ in children and adults.

| 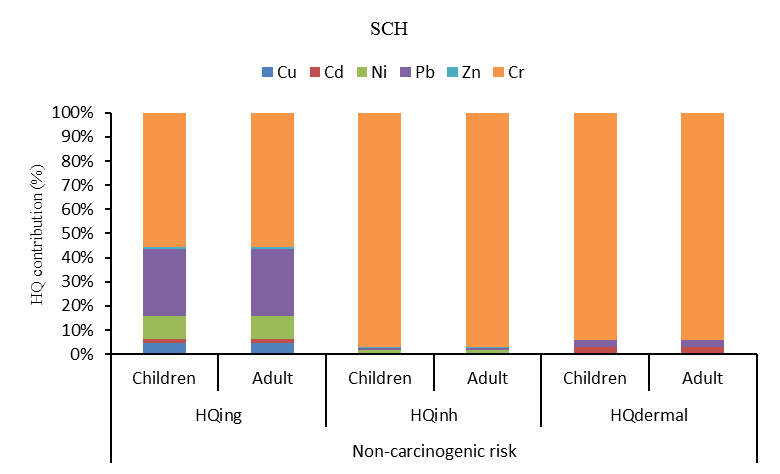 | 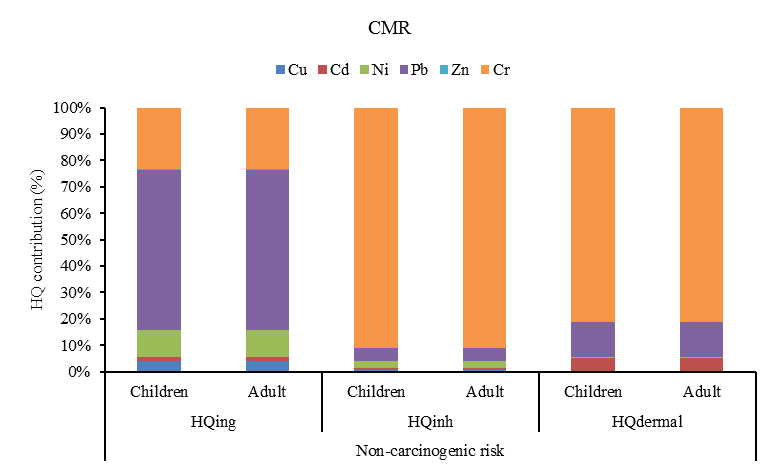 |
| --- | --- |
| 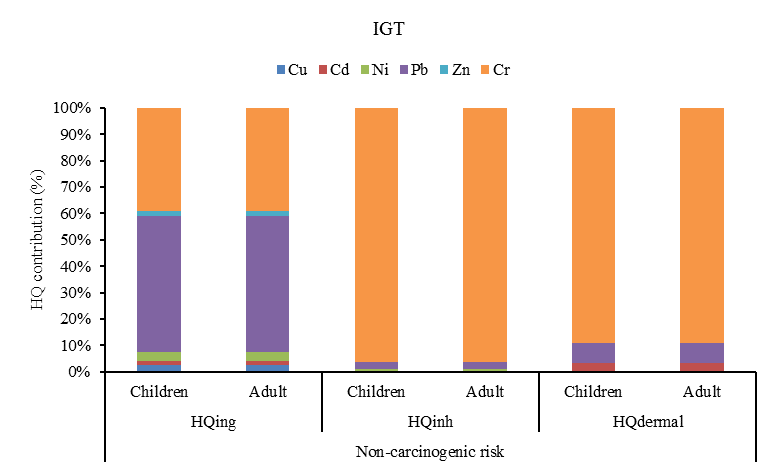 | 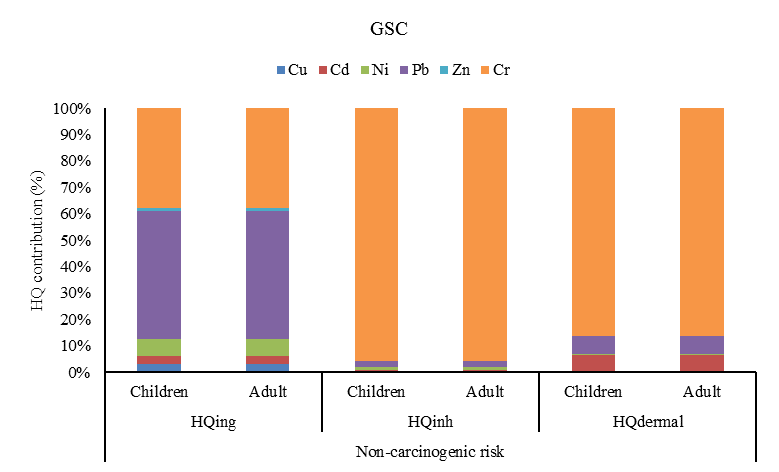 |
| 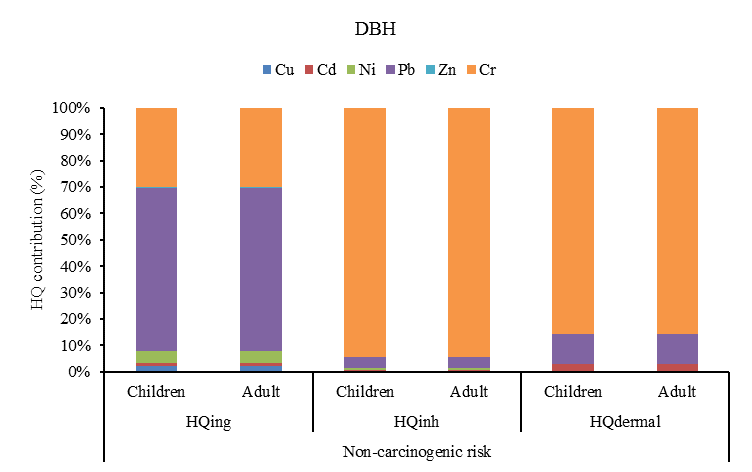 | 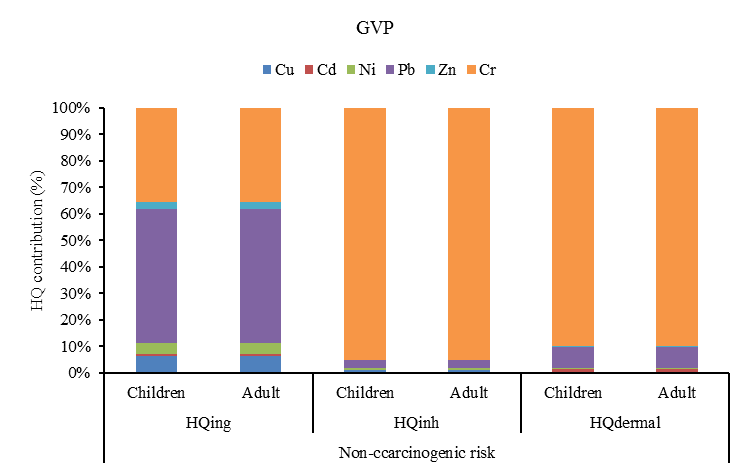 |


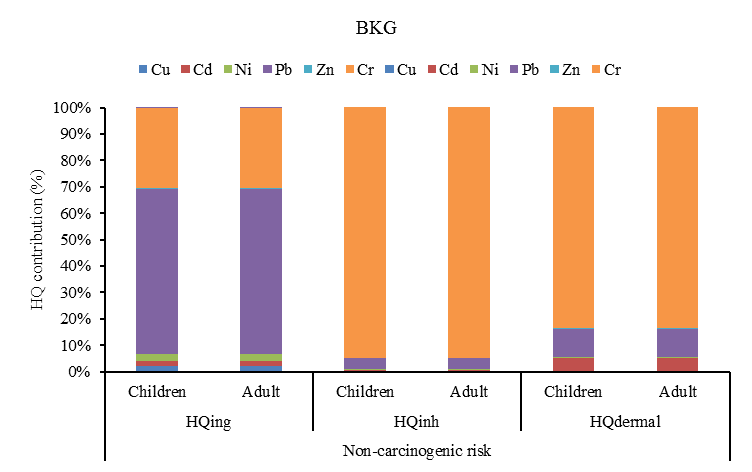


Figure S9: Hazard quotients for three pathways of exposure and carcinogenic risks posed by heavy metals in road dust in children and adults.

Table S6: Hazard Index for Three Exposure Pathways and Carcinogenic Risks Posed by Heavy Metals in PM_10_, PM_2.5_, and Road Dust in Children and Adults

|  |  | CR | | | | | |
| --- | --- | --- | --- | --- | --- | --- | --- |
| Heavy metal | Location | PM_10_ | | PM_2.5_ | | Road dust | |
|  |  | Children | Adults | Children | Adults | Children | Adults |
| Cd | SCH | 2.49288E-15 | 5.62303E-15 | 1.55879E-15 | 3.51606E-15 | 7.51E-11 | 1.69E-10 |
|  | CMR | 2.26812E-15 | 5.11606E-15 | 9.72522E-16 | 2.19366E-15 | 7.10E-11 | 1.60E-10 |
|  | IGT | 2.70525E-15 | 6.10206E-15 | 1.88441E-15 | 4.25054E-15 | 6.56E-11 | 1.48E-10 |
|  | GSC | 1.72908E-15 | 3.90018E-15 | 1.2167E-15 | 2.74444E-15 | 6.54E-11 | 1.47E-10 |
|  | DBH | 1.90201E-15 | 4.29025E-15 | 1.30188E-15 | 2.93657E-15 | 3.10E-11 | 7.00E-11 |
|  | GVP | 3.13288E-15 | 7.06665E-15 | 2.10424E-15 | 4.7464E-15 | 3.23E-11 | 7.30E-11 |
|  | BKG | 5.07764E-16 | 1.14533E-15 | 2.72284E-16 | 6.14174E-16 | 2.88E-11 | 6.50E-11 |
| Ni | SCH | 1.30665E-15 | 2.94734E-15 | 6.33557E-16 | 1.42908E-15 | 1.10E-09 | 2.49E-09 |
|  | CMR | 1.26287E-15 | 2.84857E-15 | 5.63679E-16 | 1.27146E-15 | 1.26E-09 | 2.85E-09 |
|  | IGT | 1.13939E-15 | 2.57004E-15 | 7.46533E-16 | 1.68391E-15 | 4.13E-10 | 9.32E-10 |
|  | GSC | 9.26175E-16 | 2.08912E-15 | 5.4485E-16 | 1.22899E-15 | 3.72E-10 | 8.39E-10 |
|  | DBH | 1.73262E-16 | 3.90817E-16 | 1.05826E-16 | 2.38704E-16 | 3.525E-10 | 7.9501E-10 |
|  | GVP | 9.19988E-16 | 2.07516E-15 | 6.04956E-16 | 1.36456E-15 | 6.12E-10 | 1.3805E-09 |
|  | BKG | 6.01612E-17 | 1.35702E-16 | 3.88336E-17 | 8.75946E-17 | 1.02E-10 | 2.3008E-10 |
| Cr | SCH | 3.65588E-14 | 8.24633E-14 | 2.49904E-14 | 5.63694E-14 | 4.78E-08 | 1.08E-07 |
|  | CMR | 4.5096E-14 | 1.0172E-13 | 3.4145E-14 | 7.70187E-14 | 2.17E-08 | 4.90E-08 |
|  | IGT | 5.16159E-14 | 1.16427E-13 | 2.09018E-14 | 4.7147E-14 | 3.33E-08 | 7.51E-08 |
|  | GSC | 1.25888E-14 | 2.83958E-14 | 4.34461E-14 | 9.79987E-14 | 1.64E-08 | 3.69E-08 |
|  | DBH | 3.9873E-14 | 8.99391E-14 | 1.51631E-14 | 3.42026E-14 | 1.77E-08 | 3.98E-08 |
|  | GVP | 5.86285E-14 | 1.32245E-13 | 2.01515E-14 | 4.54545E-14 | 3.78E-08 | 8.53E-08 |
|  | BKG | 5.00645E-15 | 1.12927E-14 | 2.54407E-15 | 5.73849E-15 | 8.73E-09 | 1.97E-08 |

**References**

**1.** **Sahu RR, Elumalai SP**. Identifying Speed Hump, a Traffic Calming Device, as a Hotspot for Environmental Contamination in Traffic-Affected Urban Roads. undefined. Published 2017. Accessed April 27, 2020. https://www.semanticscholar.org/paper/Identifying-Speed-Hump%2C-a-Traffic-Calming-Device%2C-a-Sahu-Elumalai/99426d0245cb39caba602c677c0f6466ba5ffdd5

**2.** **Wang F, Wang J, Han M, Jia C, Zhou Y**. Heavy metal characteristics and health risk assessment of PM2.5 in students’ dormitories in a university in Nanjing, China. *Building and Environment*. 2019;160:106206. doi:10.1016/j.buildenv.2019.106206

**3.** **Di Vaio P, Magli E, Caliendo G, et al**. Heavy Metals Size Distribution in PM10 and Environmental-Sanitary Risk Analysis in Acerra (Italy). *Atmosphere*. 2018;9(2):58. doi:10.3390/atmos9020058

**4.** **Li H-H, Chen L-J, Yu L, et al**. Pollution characteristics and risk assessment of human exposure to oral bioaccessibility of heavy metals via urban street dusts from different functional areas in Chengdu, China. *Science of The Total Environment*. 2017;586:1076-1084. doi:10.1016/j.scitotenv.2017.02.092

**5.** **Yongming H, Peixuan D, Junji C, Posmentier E**. Multivariate analysis of heavy metal contamination in urban dusts of Xi’an, Central China. *Science of The Total Environment*. 2006;355(1-3):176-186. doi:10.1016/j.scitotenv.2005.02.026

**6.** **Hu X, Ding Z, Zhang Y, et al**. Size Distribution and Source Apportionment of Airborne Metallic Elements in Nanjing, China. *Aerosol Air Qual Res*. 2013;13(6):1796-1806. doi:10.4209/aaqr.2012.11.0332

**7.** **Qadeer A, Saqib ZA, Ajmal Z, et al**. Concentrations, pollution indices and health risk assessment of heavy metals in road dust from two urbanized cities of Pakistan: Comparing two sampling methods for heavy metals concentration. *Sustainable Cities and Society*. 2020;53:101959. doi:10.1016/j.scs.2019.101959

**8.** **Lu X, Wang L, Lei K, Huang J, Zhai Y**. Contamination assessment of copper, lead, zinc, manganese and nickel in street dust of Baoji, NW China. *Journal of Hazardous Materials*. 2009;161(2-3):1058-1062. doi:10.1016/j.jhazmat.2008.04.052

**9.** **Förstner U, Müller G**. Concentrations of heavy metals and polycyclic aromatic hydrocarbons in river sediments: geochemical background, man’s influence and environmental impact. *GeoJournal*. 1981;5(5):417-432. doi:10.1007/BF02484715

**10.** **Gope M, Masto RE, George J, Hoque RR, Balachandran S.** Bioavailability and health risk of some potentially toxic elements (Cd, Cu, Pb and Zn) in street dust of Asansol, India. *Ecotoxicology and Environmental Safety*. 2017;138:231-241. doi:10.1016/j.ecoenv.2017.01.008

**11.** **Abrahim GMS, Parker RJ**. Assessment of heavy metal enrichment factors and the degree of contamination in marine sediments from Tamaki Estuary, Auckland, New Zealand. *Environ Monit Assess*. 2008;136(1-3):227-238. doi:10.1007/s10661-007-9678-2

**12.** **Tomlinson DL, Wilson JG, Harris CR, Jeffrey DW**. Problems in the assessment of heavy-metal levels in estuaries and the formation of a pollution index. *Helgolander Meeresunters*. 1980;33(1-4):566-575. doi:10.1007/BF02414780

**13.**  **Hakanson L**. An ecological risk index for aquatic pollution control.a sedimentological approach. *Water Research*. 1980;14(8):975-1001. doi:10.1016/0043-1354(80)90143-8

**14.** **Barsby A, McKinley JM, Ofterdinger U, Young M, Cave MR, Wragg J**. Bioaccessibility of trace elements in soils in Northern Ireland. *Science of The Total Environment*. 2012;433:398-417. doi:10.1016/j.scitotenv.2012.05.099

**15.** **Mirzaei R, Ghorbani H, Hafezi Moghaddas N, Martín JAR**. Ecological risk of heavy metal hotspots in topsoils in the Province of Golestan, Iran. *Journal of Geochemical Exploration*. 2014;147:268-276. doi:10.1016/j.gexplo.2014.06.011

**16.** **Wang J, Liu R, Zhang P, Yu W, Shen Z, Feng C**. Spatial variation, environmental assessment and source identification of heavy metals in sediments of the Yangtze River Estuary. *Marine Pollution Bulletin*. 2014;87(1):364-373. doi:10.1016/j.marpolbul.2014.07.048

**17.** **Wragg J, Cave M, Basta N, et al**. An inter-laboratory trial of the unified BARGE bioaccessibility method for arsenic, cadmium and lead in soil. *Science of The Total Environment*. 2011;409(19):4016-4030. doi:10.1016/j.scitotenv.2011.05.019

**18.** **Bonanno G, Lo Giudice R**. Heavy metal bioaccumulation by the organs of Phragmites australis (common reed) and their potential use as contamination indicators. *Ecological Indicators*. 2010;10(3):639-645. doi:10.1016/j.ecolind.2009.11.002

**19.** **Liu X, Zhai Y, Zhu Y, et al**. Mass concentration and health risk assessment of heavy metals in size-segregated airborne particulate matter in Changsha. *Science of The Total Environment*. 2015;517:215-221. doi:10.1016/j.scitotenv.2015.02.066

**20.** **Men C, Liu R, Xu F, Wang Q, Guo L, Shen Z**. Pollution characteristics, risk assessment, and source apportionment of heavy metals in road dust in Beijing, China. *Science of The Total Environment*. 2018;612:138-147. doi:10.1016/j.scitotenv.2017.08.123

**21.**  **Li H, Qian X, Hu W, Wang Y, Gao H**. Chemical speciation and human health risk of trace metals in urban street dusts from a metropolitan city, Nanjing, SE China. *Science of The Total Environment*. 2013;456-457:212-221. doi:10.1016/j.scitotenv.2013.03.094

**22.** **Kong S, Lu B, Ji Y, et al**. Levels, risk assessment and sources of PM10 fraction heavy metals in four types dust from a coal-based city. *Microchemical Journal*. 2011;98(2):280-290. doi:10.1016/j.microc.2011.02.012

**23.** **Hu X, Zhang Y, Ding Z, et al**. Bioaccessibility and health risk of arsenic and heavy metals (Cd, Co, Cr, Cu, Ni, Pb, Zn and Mn) in TSP and PM2.5 in Nanjing, China. *Atmospheric Environment*. 2012;57:146-152. doi:10.1016/j.atmosenv.2012.04.056

**24.** **Ferreira-Baptista L, De Miguel E**. Geochemistry and risk assessment of street dust in Luanda, Angola: A tropical urban environment. *Atmospheric Environment*. 2005;39(25):4501-4512. doi:10.1016/j.atmosenv.2005.03.026

**25.** **U.S. Environmental Protection Agency**. Risk Assessment Guidance for Superfund Volume I: Human Health Evaluation Manual (Part E, Supplemental Guidance for Dermal Risk Assessment). Office of Superfund Remediation and Technology Innovation, Washington, D.C. 2004.
